# Effect of aquatine endodontic cleanser on smear layer removal in the root canals of ex vivo human teeth

**DOI:** 10.1590/S1678-77572010000400014

**Published:** 2010

**Authors:** Faustino GARCIA, Peter E. MURRAY, Franklin GARCIA-GODOY, Kenneth N. NAMEROW

**Affiliations:** DDS, Graduated resident (Endodontics), Private Practice, Formerly of the Department of Endodontics, College of Dental Medicine, Nova Southeastern University, Fort Lauderdale, Florida, USA.; BSc(Hons), PhD, Professor, Department of Endodontics, College of Dental Medicine, Nova Southeastern University, Fort Lauderdale, Florida, USA.; DDS, MS, Professor and Executive Associate Dean for Research, College of Dentistry, University of Tennessee, Memphis, USA.; DDS, Associate Professor, Director of Postgraduate Endodontics, and Chair, Department of Endodontics, College of Dental Medicine, Nova Southeastern University, Fort Lauderdale, Florida, USA.

**Keywords:** Irrigation, Dental pulp, EDTA, Sodium hypochlorite, Hypochlorous acid

## Abstract

**Objectives:**

The purpose of this study was to measure and compare the root canal cleanliness and smear layer removal effectiveness of Aquatine Endodontic Cleanser (Aquatine EC) when used as an endodontic irrigating solution in comparison with 6% sodium hypochlorite (NaOCl).

**Material and Methods:**

Forty-five human teeth were randomly allocated to five treatment groups; the pulp chamber was accessed, cleaned, and shaped by using ProTaper and ProFile rotary instrumentation to an ISO size #40. The teeth were then processed for scanning electron microscopy, and the root canal cleanliness and removal of smear layer were examined.

**Results:**

The most effective removal of smear layer occurred with Aquatine EC and NaOCl, both with a rinse of EDTA.

**Conclusions:**

Aquatine EC appears to be the first hypochlorous acid approved by the FDA to be a possible alternative to the use of NaOCl as an intracanal irrigant. Further research is needed to identify safer and more effective alternatives to the use of NaOCl irrigation in endodontics.

## INTRODUCTION

Cleaning and disinfection are the main objectives of root-canal preparation. Thorough cleaning removes microorganisms, permits adaptation of filling materials and enhances the action of intracanal medicaments. The choice of an irrigant is of great importance because they act as lubricants during instrumentation, flush debris and bacteria out of the canal, and react with pulp, necrotic tissues and microorganisms. Numerous irrigants have been recommended for clinical use^16^. Irrigation with distilled water is effective at removing loose debris, but has little effect on smear layer or microorganisms^1^. Sodium hypochlorite (NaOCl) has been extensively used as an irrigating solution for several decades, and it is widely recommended^9^. Its excellent properties of tissue dissolution and antimicrobial activity make it the irrigant of choice for the treatment of teeth with pulp necrosis^19^, even though it has several undesirable characteristics such as tissue toxicity at high concentrations, risk of emphysema when overfilling, and allergic potential^21^. Moreover, NaOCl does not totally clean the surfaces of the root canals^1^. These problems suggest that NaOCl irrigation is not fully optimized and there is a need to identify irrigants which are effective, but also biocompatible, to avoid the risk of harming patients.

The smear layer is a 1-5 mm thick layer^2^ of denatured cutting debris produced on instrumented cavity surfaces, and is composed of dentin, odontoblastic processes, non-specific inorganic contaminants and microorganisms^5^. The removal of smear layer from the instrumented root canal walls is controversial^17^. Its removal provides better sealing ability of the endodontic filling material to dentin, thereby avoiding leakage of microorganisms into the oral tissues^23^. The infiltration of microorganisms into oral tissues must be prevented because it is believed these often cause complications leading to treatment failure.

The disinfection of root canals through the elimination of microorganisms is an essential step in endodontic treatment^3^ to help avoid subsequent failure^10^. Surface adherence by bacteria to form biofilms is a good example of bacterial adaptation and one that is pertinent to endodontic infections. Increasing information is now available on the existence of biofilm communities on root canal walls^28^. Unfortunately, complete disinfection is difficult to accomplish; microorganisms can remain within the apical dentin plug^15^, within the smear layer^8^ and within the dentinal tubules^18^. To maximize the removal of microorganisms, the shaping and mechanical enlargement of a root canal must be accompanied by copious irrigation^10,13^. The ideal irrigant should have an antimicrobial action, low toxicity and good biocompatibility to oral tissues. In addition, it should have the capacity to clean the walls of the root canal and remove the smear layer.

In August 2006, the U.S. Food and Drug Administration cleared Sterilox Dental’s Aquatine endodontic Cleanser (Aquatine eC, Sterilox Puricore, Malvern, PA, USA) for use as an endodontic irrigating solution. Aquatine eC is intended to irrigate, cleanse and debride the root canal system (510k number K061689). The active component in Aquatine eC is hypochlorous acid (HOCl). HOCl is produced by the body’s immune cells, via a chain of aerobic reactions called the Oxidative Burst Pathway, to kill invading pathogens and to fight infection^6^. Sterilox Puricore (Malvern, PA, USA) has developed a range of medical products that contain different concentrations of HOCl. The HOCl solution is produced by electrochemically charging a low concentration salt solution using an element reactor. HOCl is commonly used for hospital disinfection, sterilization, and in the treatment of chronic wounds^22,25^. In dentistry it is commonly used to disinfect water lines by removing biofilms^7,12^. HOCl is biocompatible and antimicrobial against a broad range of microorganisms^12^. A pilot study of the cleaning effectiveness of an electrochemically activated solution (EAS) that contained a mixture of HOCl, ClO^-^, ClO and H_2_O_2_ at pH 7.7 found its smear layer removal effectiveness was superior to 3% NaOCl27. However, there have been no studies to date examining the effectiveness of Aquatine EC as an endodontic irrigant.

The objective of this study was to evaluate Aquatine eC as an endodontic irrigating solution in a simulated clinical setting where bacterial invasion of the dental tubules occurs prior to biomechanical instrumentation. The cleaning effectiveness of Aquatine EC to remove bacterial biofilm, debris and smear layer from root canals was assessed by visualization, using scanning electron microscopy.

## MATERIAL AND METHODS

A pre-existing archive of extracted human teeth was used for this study following institutional review board approval. The intact, randomly selected, permanent teeth had not previously received any root canal medicaments nor were any stored in antibacterial or fixative solutions. The teeth were X-rayed prior to inclusion in this project to ensure that all the teeth had a single root canal, and the root lengths were approximately 18 mm. The teeth were de-crowned at the cementoenamel junction using a diamond rotary bone-cutting saw (Materials Science, NW Ltd, Settle, england, UK). each tooth was placed in an eppendorf tube and filled with brain heart infusion broth (BHI, Difco Laboratories, Detroit, MI, USA).

Pure culture *E. faecalis* (ATCC 29212, PML Microbiologicals, Wilsonville, OR, USA) grown in BHI broth (Difco Laboratories) was used to contaminate the eppendorf tubes containing the extracted teeth. each individual tooth was inoculated with 10 mL of a 1.5x10^8^ CFU mL^-1^ suspension using a sterile 1 mL using a tuberculin syringe. The teeth were incubated in a 5% CO_2_ atmosphere at 37ºC for 28 days^4,24^. During the 28 day infection period, the BHI media was refreshed every third day to ensure bacterial viability. After 28 days of *in vitro* culture in the presence of e. faecalis, the absorbance of the BHI culture media was measured at 600 nm to ensure that all (100%) of the teeth were contaminated^20^ prior to the shaping and cleaning of root canals. At 28 days, the external and internal surfaces of each tooth were sampled with sterile fine paper points and inoculated on BHI agar plates to confirm infection of the specimens. *E. faecalis* in pure culture was determined by visualization of individual white pinpoint colonies on the BHI agar plates. Confirmation was determined by microscopic observation of Gram-positive cocci arranged in a cross-chain pattern, following the protocol described by Shabahang and Torabinejad^24^ (2003).

The teeth were instrumented with ProTaper (Dentsply Tulsa Dental, Oklahoma City, OK, USA) file series to F3, and the canals were further enlarged with Profiles (Dentsply, Tulsa Dental) 35/.06 and 40/.06 similar to the methods described by Shabahang and Torabinejad^24^ (2003), and Crumpton, Goodell and McClanahan^4^ (2005). The working length was determined by passively placing a #10 K-file (Dentsply Tulsa Dental) in the canal until the tip of the instrument visibly penetrated and was adjusted to the apical foramen. The actual canal length was measured, and the working length was calculated by subtracting 1 mm from this measurement. During cleaning and shaping, 5 mL of irrigating solution was used with each instrument size. In each canal during instrumentation, a total volume of 25-30 mL of irrigation solution was delivered using small plastic needles (Ultradent Products, South Jordan, UT, USA). The following irrigation procedures were used: group i) Control group: The canals of 5 instrumented teeth were irrigated with distilled water to serve as negative controls. The remaining 40 teeth were divided into 4 experimental groups of 10 teeth per group: group ii), the root canals were irrigated during instrumentation with Aquatine eC solution. The Aquatine eC hypochlorous acid (HOCl) solution (180-250 ppm of available free chlorine (AFC), pH 5.35-6.75) was prepared fresh, by electrolysis, immediately prior to use. The concentration of (AFC) was measured photometrically following the acidification of the Hypochlorous acid [HOCl] to Chlorine [Cl^-^] (Palintest Inc., KY, US). Group iii), the root canals were irrigated with Aquatine eC solution as described in group ii), followed by the application of 2 mL of 17% eDTA for 15 s^26^. Group iv), the root canals were irrigated with 6% NaOCl (Clorox, Oakland, CA, USA). In group v), the root canals were irrigated with 6% NaOCl followed by the application of 2 mL of 17% EDTA (PulpDent, Watertown, MA, US) for 15 s. These methods were congruent with those of Shabahang and Torabinejad^24^ (2003).

The effectiveness of the irrigation treatments to clean the root canals were assessed using micrograph images of the root canals collected using a scanning electron microscope (SeM). The teeth were prepared for use in the SEM by fixing the tooth tissues in 10% neutral-buffered formalin solution at 18°C for 24 h. The teeth were then post-fixed in osmium tetroxide (1% w/v) for 2 h before being dehydrated in a graded series of ethanol solutions. The teeth were dried on filter paper for 24 h and then fractured longitudinally along the length of the canal using a chisel. each tooth-half was mounted onto aluminum stereoscan stubs with carbon tape (Ted Pella Inc., Redding, CA, USA) with the entire length of the root canal visible and facing upwards. each of the specimens was coated with a 20-30 nm thin metallic layer of gold/palladium in a Polaron e5000 sputter coater (BioRad, Hercules, CA, USA). The samples were viewed in a Quinta 200 SeM (FeI, Hilsboro, OR, USA). SeM micrographs were obtained at x2,000 magnification using digital image analysis software. each of the root canals was scanned in its entirety to obtain an overview of the general surface topography^24^. Micrographs were taken of representative areas characteristic of the general surface topography of each specimen, including the apical, middle and coronal aspects^14^. The dentin root canal surfaces were assessed for the presence of smear layer by two double-blind reviewers using semi-quantitative visual criteria described by Crumpton, Goodell and McClanahan^4^ (2005), Madison and Hokett^11^ (1997) and Tay, et al.^29^ (2006) using a 4-step scale: (0) All tubules visible. (1) More than 50% of tubules visible. (2) Less than 50% of tubules visible, and (3) No tubules visible. The removal of smear layer from the root canals was analyzed using Chi-Square (χ^2^) statistics tests (Statview, SPSS, Cary, NC, USA).

## RESULTS

After 28 days of *in vitro* culture in the presence of *E. faecalis*, the absorbance counts from the BHI culture media of each tooth gave high absorbance counts at 600 nm, indicating that all (100%) of the teeth were contaminated^20^ prior to the shaping and cleaning of root canals.

The available free chlorine (AFC) concentration of the Aquatine eC was tested prior to each use, by measuring the hypochlorous acid content; it was stably produced by electrolysis (Sterilox Dental, Malvern, PA, USA) at a concentration of 180-200 ppm AFC, pH 6.0.

Analysis of the smear layer removal data for differences between the coronal, middle and apical aspect of teeth found no significant differences (χ^2^, p>0.05). Therefore, the data was not stratified according to the different aspects of teeth prior to further statistical analysis.

**Figure 1:**
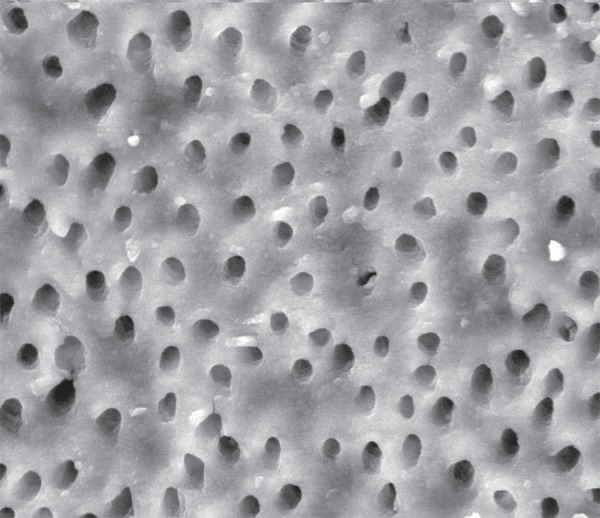
Scanning Electron Microscopy (SEM) micrograph of a root canal irrigated with Aquatine EC and a rinse of EDTA. All dentinal tubules are visible and the smear layer was completely removed

**Figure 2:**
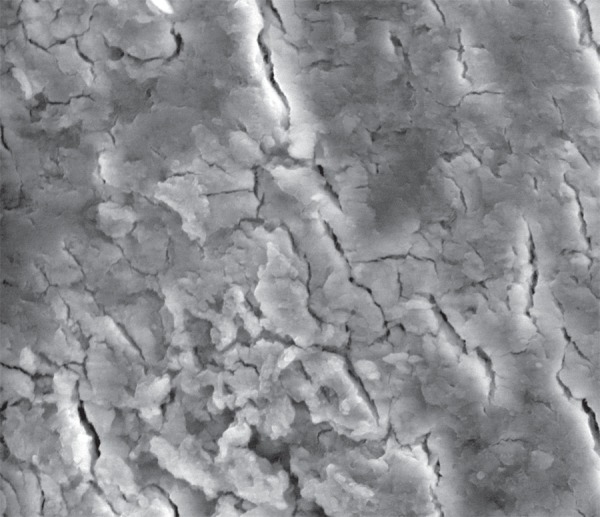
Scanning Electron Microscopy (SEM) micrograph of the smear layer in root canals irrigated with distilled water. No dentinal tubules are visible because of the smear layer covering the root canal surface

**Figure 3:**
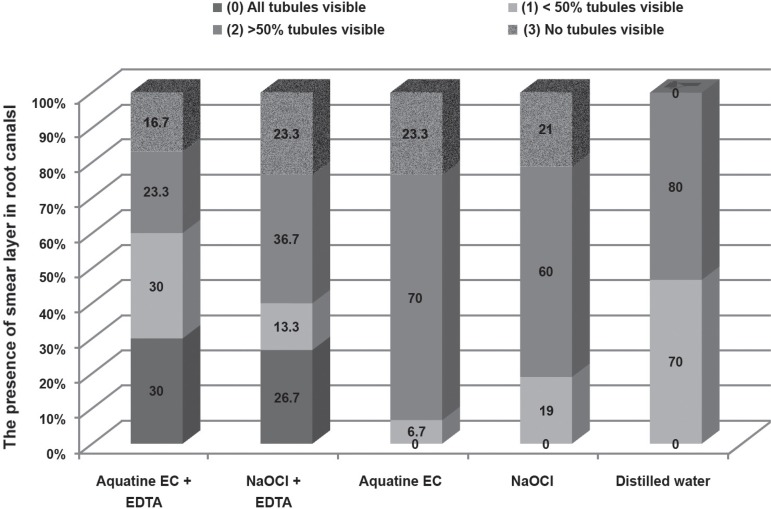
Presence of smear layer in root canals following root canal irrigation treatments. The presence of smear layer criterion is shown as a percentage of root canals for each of the irrigation treatments

**Figure 4:**
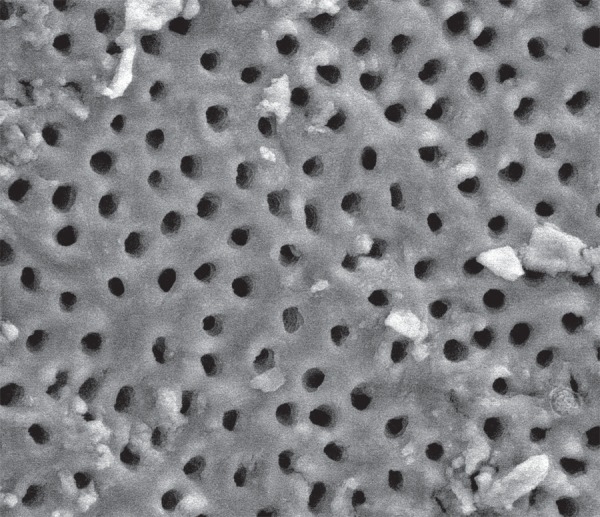
Scanning Electron Microscopy (SEM) micrograph of the smear layer in root canals irrigated with NaOCl and a rinse of EDTA. All dentinal tubules are visible and the smear layer was completely removed

**Figure 5:**
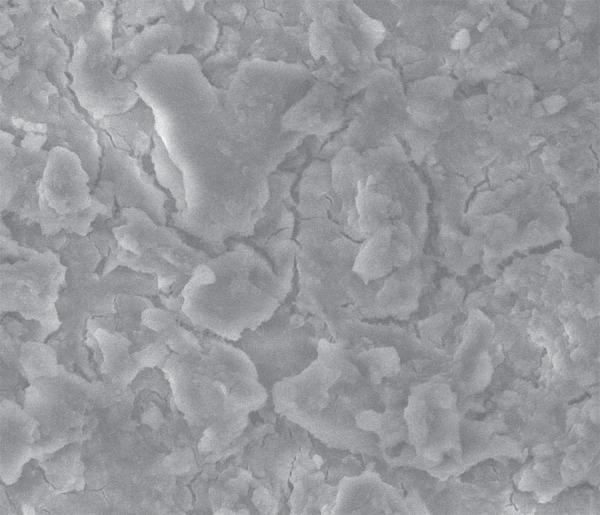
Scanning Electron Microscopy (SEM) micrograph of a root canal irrigated with Aquatine EC. No dentinal tubules are visible because of the smear layer covering the root canal surface

**Figure 6:**
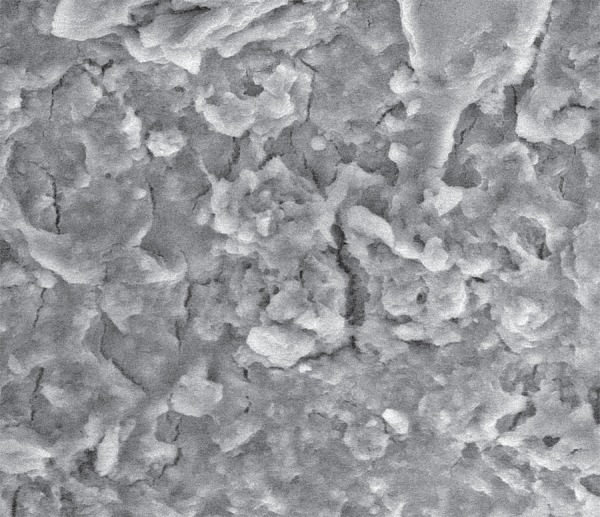
Scanning Electron Microscopy (SEM) micrograph of the smear layer in root canals irrigated with NaOCl. No dentinal tubules are visible because of the smear layer covering the root canal surface

The removal of smear layer covering dental tubules was influenced by the use of different irrigation treatments (χ^2^, p<0.05). The most complete removal (100%) of smear layer covering root canal dentinal tubules was observed following root canal irrigation with Aquatine eC and a rinse of eDTA (Figure 1). The least removal of smear layer covering root canal dentinal tubules was observed following irrigation with distilled water (Figure 2) which was a control group. Aquatine eC and eDTA completely removed the smear layer in 30% of teeth, and removed more than half the smear layer in a further 30% of teeth (Figure 3). NaOCl and eDTA (Figure 4) completely removed 26.7% of the smear layer covering dentinal tubules, and more than half the smear layer covering the dentinal tubules in a further 13.3% of teeth (Figure 3). Although there were more dentinal tubules covered with smear layer following NaOCl irrigation with a rinse of eDTA (Figure 3), there was little difference in comparison with Aquatine eC followed by a rinse of eDTA (χ^2^, p>0.05). The greatest presence of smear layer was observed covering dentinal tubules in the root canals irrigated with Aquatine eC (Figure 5) and NaOCl (Figure 6) without eDTA (Figure 3), which were control groups.

## DISCUSSION

The removal of smear layer from root canals appeared to be influenced by the selection of endodontic irrigants and the use of eDTA during root canal instrumentation. A previous *in vitro* study has demonstrated the effectiveness of HOCl to disinfectant mixed species biofilms^30^, and that it has excellent biocompatibility to tissues. The biocompatibility and safety of Aquatine eC have earned it FDA clearance as a medical device in late 2006 to be sold and marketed as an endodontic irrigant. This product is new, and it has not been previously tested as an endodontic irrigant. Only one previous pilot study of 20 teeth by Solovyeva and Dummer^27^ (2000) has been published about the cleaning effectiveness of a pH 7.7 eAS containing a mixture of biological reagents including HOCl. That study, observed the eAS in comparison to NaOCl, removed more smear layer and more debris, leaving cleaner canals. Because of these beneficial results, Solovyeva and Dummer^27^ (2000) have advocated eAS to be used as an alternative to NaOCl, but no further progress to advance the introduction of eAS into endodontic practice appears to have been made, until now.

NaOCl is recommended for use as an endodontic irrigant by the American Association of endodontists, but it is not approved by the FDA because of its high toxicity, caustic hazard, risk of emphysema in case of overfilling, and the severe allergic reactions that can result in patient suffering^21^. The widespread use of NaOCl as an endodontic irrigating solution can be explained by its low price, excellent necrotic pulp tissue dissolution properties, and its excellent root canal disinfection properties. Since NaOCl is the endodontic "gold standard", experimental irrigants must be compared with it, to be able to compare smear layer removal. The comparison between NaOCl and Aquatine eC in this present study found that when both are used with a rinse of eDTA; they are similarly effective at removing debris and cleaning root canals, and removing smear layer covering dental tubules and also inside the dental tubules.

In the present study, the root canals were contaminated with *E. faecalis* to allow for ease of maintenance and identifying the growth of a single species. A 28-day infection period allowed for biofilm growth and the penetration of bacteria into the dentinal tubules^24^. The microbial sampling demonstrated that bacteria remained viable throughout the experiment. Most studies of bacterial growth from root canals have visualized turbidity in the culture media as the end point. In order to avoid subjectivity in determining turbidity, visual assessment we used, and the absorbance of the BHI culture was measured in a spectrophotometer.

The presence of smear layer prevents penetration of antibacterial agents into the dentinal tubules, indicating that its removal may benefit disinfection and also sealing and adhesion of endodontic sealers to root canal walls^29^. The previous pilot study of eAS demonstrated its ability to partially remove smear layer in the absence of a chelating agent^27^. The present results are somewhat in agreement, but showed that smear layer removal was more optimal in the Aquatine eC teeth with a rinse of 17% eDTA, compared to teeth instrumented without eDTA. In the absence of a rinse of eDTA, the Aquatine eC was not very effective at cleaning root canals or removing smear layer, indicating that for optimal performance, Aquatine eC must be used with a rinse of eDTA. It is unlikely that the length of time, or quantity of eDTA used for irrigation causes marked differences between different studies^30^. This indicates that any differences observed in smear layer removal are caused by the difference between the eAS in the pilot study^27^ and this present study. The potency of the Aquatine eC was tested prior to each use, by measuring the HOCl content; it was stably produced by the electrolysis unit at a concentration of 180-200 ppm AFC, pH 6.0. In the pilot study the anolyte neutral cathodic solution - had an active chlorine concentration of 300 mgL^-1^. Indicating that the eAS was slightly different in composition to Aquatine eC and likely was more potent. This difference serves to demonstrate that both eAS and Aquatine eC may be able to remove smear layer without chelating agents if the concentration of hypochlorous acid is increased.

## CONCLUSIONS

On the basis of these results, it appears that Aquatine eC has a similar effectiveness as NaOCl when used with a rinse of eDTA to clean root canals of debris and to remove smear layer following contamination with *E. faecalis.* The cleanliness of the root canals and the degree of smear layer removal were comparable with that of 6% NaOCl. Aquatine eC may be superior to NaOCl in terms of safeguarding patients from accidents because it is a biocompatible root canal cleanser, whereas NaOCl is not. Aquatine eC could therefore provide a safer alternative to NaOCl disinfection for the removal of biofilm bacteria in endodontic canals. Further studies are needed to determine the effect of these findings in clinical settings.
